# Differential, Positional-Dependent Transcriptional Response of Antigenic Variation *(var)* Genes to Biological Stress in *Plasmodium falciparum*

**DOI:** 10.1371/journal.pone.0006991

**Published:** 2009-09-10

**Authors:** Elli Rosenberg, Amir Ben-Shmuel, Oshrit Shalev, Rosa Sinay, Alan Cowman, Yaakov Pollack

**Affiliations:** 1 The Shraga Segal Department of Microbiology and Immunology, Ben-Gurion University of the Negev, Beer-Sheva, Israel; 2 Department of Infection and Immunity, The Walter & Eliza Hall Institute of Medical Research, Melbourne, Australia; New Mexico State University, United States of America

## Abstract

1% of the genes of the human malaria causing agent *Plasmodium falciparum* belong to the heterogeneous *var* gene family which encodes *P. falciparum* erythrocyte membrane protein 1 (PFEMP1). This protein mediates part of the pathogenesis of the disease by causing adherence of infected erythrocytes (IE) to the host endothelium. At any given time, only one copy of the family is expressed on the IE surface. The cues which regulate the allelic exclusion of these genes are not known. We show the existence of a differential expression pattern of these genes upon exposure to biological stress in relation to their positional placement on the chromosome – expression of centrally located *var* genes is induced while sub-telomeric copies of the family are repressed - this phenomenon orchestrated by the histone deacetylase *pfsir2*. Moreover, stress was found to cause a switch in the pattern of the expressed *var* genes thus acting as a regulatory cue. By using pharmacological compounds which putatively affect *pfsir2* activity, distinct changes of *var* gene expression patterns were achieved which may have therapeutic ramifications. As disease severity is partly associated with expression of particular *var* gene subtypes, manipulation of the IE environment may serve as a mechanism to direct transcription towards less virulent genes.

## Introduction

The mechanisms that have evolved for the exclusive expression of a particular gene from a multi-gene family differ between various organisms and cells, examples being the expression-linked site in the trypanosome antigenic variation VSG family [1], the B-cell/TCR rearrangement machinery [2] and the olfactory receptor repertoire [3]. *P. falciparum* harbors several such multi-gene families [4]. Of these, the most widely studied one, the *var* gene family, encodes PFEMP1 - a polymorphic molecule of both clinical importance and of great scientific interest as its regulatory mechanism is unlike any other system involved in allelic exclusion [5].

During the erythrocytic stage of its lifecycle, *P. falciparum* exports a plethora of proteins to the surface of the IE, many of these are involved in the modification of the IE cytoskeleton and membrane to create the characteristic knob structure on the IE surface [6]. PFEMP1 protrudes from the knob to engage with various endothelial receptors, such as ICAM-1, CD36 and CSA, causing the cytoadherence of the IE to the endothelial cells and its sequestration in the microvasculature - the dire clinical complications of this disease, such as cerebral malaria and placental malaria, are the consequence of this phenomenon [7]. PFEMP1 is encoded by the 59 member *var* gene multifamily, and it is accepted that in an individual parasite, at any given moment, only one *var* gene is expressed [8]–[10]. This exclusively expressed gene can be switched to an alternative member of the *var* repertoire thereby achieving antigenic variation and effectively avoiding host immune system clearance. The *var* genes are divided into three main subtypes (*upsA*, *upsB* and *upsC*), based on their 5′ upstream promoter sequences [4], [11], [12]. *UpsA*-type *var* genes are located in subtelomeric regions, *upsB*-type *var* genes are located subtelomerically with many of them immediately adjacent to the telomeres and *upsC*-type *var* genes are found in chromosome-internal clusters. Interestingly, these subtypes have a clear clinical significance as disease phenotype is linked to the subtype expressed. For instance, severe malaria was correlated with the expression of *upsA* [13] or *upsB* [14] or both [15] *var* gene subtypes whereas asymptomatic malaria was correlated with the *upsC* subtype [14]. The regulation of *var* gene expression and the mechanisms responsible for maintenance of allelic exclusion occur at the transcriptional level [16]. Various multilayered epigenetic mechanisms have been implicated in this transcriptional control [17]: perinuclear locus repositioning of a *var* gene to a transcriptionally active site [18], [19], repression activity of the intron of the expressed *var* gene [20]–[22] and modification of chromatin structure [23]–[25]. In regard to the latter, an orthologue of the *S. cerevisiae* NAD^+^ dependent histone deacetylase (HDAC) *sir2* (Silencing Information Regulator 2, also known as sirtuin), involved in alteration in chromatin structure was identified in *P. falciparum* and termed *pfsir2* [24]. It was shown to associate with the telomeric *rep20* sequences and induce deacetylation of histone H4 complexes thus spreading gene repression ∼50 kb into chromosome internal regions, regions that include many *var* genes. Additionally, it was found to be released upon *var* gene activation concomitantly with acetylation of histone H4. This data implicates *pfsir2* and the telomere positional effect (TPE) as central players in *var* gene regulation. Interestingly, an additional plasmodial HDAC, termed *psir2B*, was recently found to exclusively suppress expression of the *upsB* subype [26].

While the data which accumulated in recent years open new doors in our understanding of *var* gene regulation and allelic exclusion, several questions still remain unanswered. Is the selection of a particular *var* gene random or is it dependent on other cellular conditions? More specifically, what are the cues involved in determination of the *var* positioned for exclusive expression? Finally, does the expression of certain *var* genes confer a survival advantage to the parasite?

We hypothesized that transcription of *var* genes, as encoders of an external protein which interacts with its surroundings, may be differentially affected by environmental conditions the parasite encounters throughout it's complex life-cycle.

## Materials and Methods

### Parasite culturing and synchronization

The *in vitro* culturing and synchronization of *P. falciparum* NF-54 isolate was carried out by standard protocols as described previously [27]. Briefly, the parasites were cultured in flasks at 37°C and 5% hematocrit in RPMI 1640 medium supplemented with human plasma (A^+^ or AB^+^), 50 µg/ml gentamycin, 25 mM HEPES, and 0.25% sodium bicarbonate in an atmosphere of 5% O_2_, 5% CO_2_ and 90% N_2_. When cultures exceeded parasitemia of 10%, they were diluted to 3%. Prior to experimentation, parasite cultures were synchronized to early ring stage using 5% sorbitol, the procedure preformed twice consecutively ensuring removal of all parasites not at the ring stage.

We used two parasite lines which were genetically manipulated. The first, 3D7/upsC, is a 3D7 parasite carrying a pHBupsC plasmid as an episome [21]. This plasmid contains genes coding for blasticidin deaminase (*bsd*) and human dihydrofolate reductase (h*dhfr*), conferring resistance to blasticidin-S and WR99210 (WR) respectively. The transfected parasites were grown with 2 µgml^−1^ blasticidin-S added to the culture medium to obtain parasites carrying episomes, and then continuously exposed to 4 nM WR for at least 40 generations to guarantee selection of parasites exclusively expressing the episomal h*dhfr*. The second line used is a *pfsir2* knockout (Δ*pfsir2*) [23].

### Stress induction

Ring-stage parasites (6 hours post synchronization) were exposed to two forms of stress inducing conditions: oxidative stress (10 µM of *tert*-butylhydroperoxide [tBHP] for 4 hours) and glucose deprivation (4 hour culture in the presence of 1.95 g/l glucose, 50% of the normal concentration), immediately followed by RNA extraction. The timing of this analysis at 10 hours post synchronization coincides with the peak of *var* gene expression determined by a time-curve assay (data not shown). This was performed by qPCR analysis of total *var* gene expression using *var* universal primers at 10, 14 and 24 hours post-synchronization. The stress conditions used in these experiments did not affect the vitality and growth of the parasites as judged by a hypoxanthine incorporation assay and examination of parasitemia levels.

### Pharmacological agents

The effect of two compounds was examined – resveratrol (RV) and *N*-acetylcysteine (NAC). A 1 M stock solution of RV was prepared in DMSO from powder (Sigma) which was further diluted in double-distilled water. RV was freshly prepared for each experiment. NAC, supplied as liquid (CellTech) was diluted in double-distilled water. Synchronized ring stage parasites were exposed to either agent at 2 hours post synchronization for 8 hours with RNA extraction at 10 hours post synchronization.

### Hypoxanthine incorporation assay

At 18 hours post-stress induction, parasites were cultured for 18 hours in a 96 well plate at a hematocrit of 1.5% and 1% parasitemia in the presence of [^3^H]hypoxanthine (0.5 µCi per well). Cells were harvested using an Inotech cell harvester and radioactivity counted in a β-scintillation counter.

### RNA extraction and cDNA synthesis

Free parasites were obtained from the IE by lysis with 0.05% saponin and pelleted by centrifugation. Purification of total RNA from parasite pellets was carried out using Tri-Reagent in accordance with the manufacturer's protocol. Carry-over DNA was eliminated by DNase treatment (Turbo DNA-free, Ambion) and its absence was validated by PCR using primers for the 18S ribosomal RNA gene. Only completely DNA-free total RNA samples were used for cDNA synthesis (ABgene ConvertIT kit in accordance with the protocol supplied by the manufacturer) using random decameres as primers.

### Gene expression analysis

Relative gene expression was calculated using the quantitative real-time PCR (qPCR) method with primers designed for the conserved *upsA*, *B* and *C* 5′ upstream sequences [14], individual *var* genes [28], 18S ribosomal subunit (forward 5′-GCTGACT-ACGTCCCTGCCC-3′; reverse 5′-ACAATTCATCATATCTTTCAATCGGTA-3′) the seryl *t*RNA synthetase (forward 5′-TGGAACAATGGTAGCTGCACAAA-3′; reverse 5′-ATGGGCGCAATTTTTCAGGA-3′) and for h*dhfr* (forward 5′- GAATCACCC-AGGCCATCTTA-3′; reverse 5′- GCCTTTCTCCTCCTGGACAT-3′). qPCR was performed on the ABgene thermocycler platform with the ABsolute SYBRgreen ROX mix (ABgene), 1 ng of cDNA template and 100 nM gene specific primers. The 18S ribosomal subunit and the seryl *t*RNA synthetase genes were used as endogenous controls. These primers and the primer for h*dhfr* were examined for efficiency, displaying an amplification slope of −3.33±0.3 and r^2^≥0.98. Data analysis was performed using the ΔΔC_T_ method with the ABgene thermocycler software. Expression analysis experiments were repeated between 3 to 6 times, each experiment in triplicates.

## Results

### Effect of environmental stress on the expression of var genes

**Figure 1:**
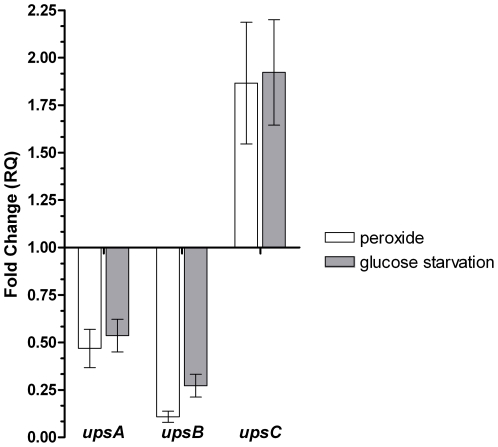
The relative change in expression of the *ups A, B*, and *C var* subtypes upon exposure to stress in NF-54 parasites. Synchronized ring stage parasites were exposed to a 4 hour pulse of stress inducing conditions (either 10 µM of *tert*-butylhydroperoxide or cultivation in the presence of 50% of the normal glucose concentration). cDNA prepared from RNA isolated from these parasites was analyzed utilizing qPCR with *ups*-specific primers. Results are expressed as fold change in comparison to an unexposed control (Relative Quantification, RQ). Error bars represent mean ± SEM.

Synchronized NF-54 ring stage parasites at 6 hours post-synchronization were exposed to two forms of stress: oxidative, via exposure to tBHP and nutritional deprivation by culturing the parasite in the presence of half of the normal glucose concentration. Stress was applied for 4 hours immediately followed by total RNA extraction. The change in *ups var* gene subtype expression was examined utilizing qPCR with general primers for the *upsA*, *B*, and *C* subtypes. The results presented in Fig. 1 show the change in the *ups* subtype expression pattern upon exposure to the two forms of stress. Subtelomeric *upsB* subtype expression is repressed to the highest extent, with a more moderate repression of subtelomeric *upsA* subtype expression. This contrasts the definite induction of expression of the *upsC* subtype located in the central regions of the chromosome.

**Figure 2:**
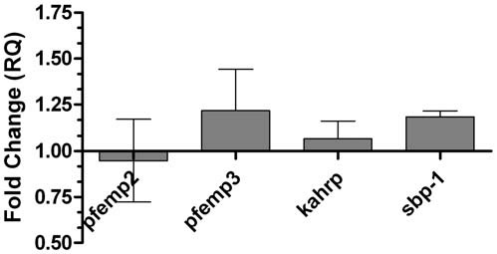
Analysis of expression of exported non-*var* genes upon exposure to 10 µM tBHP in NF-54 parasites. Stress induction, cDNA production and qPCR analysis were performed as described in the legend to Fig. 1. Error bars represent mean of triplicates ± SEM.

In order to examine whether this seemingly positional pattern is unique to *var* genes or whether it is a general positional-dependent pattern, we examined the effect of stress on other exported non-*var* genes. The genes chosen are all placed at various distances from the telomeres and are all exported to compartments unique to an IE - either the knob structure (PFEMP2, PFEMP3, KAHRP) or the Maurer's cleft (SBP-1) [29]. SBP-1, PFEMP3 and KAHRP are also involved in the positioning of PFEMP1 on the IE membrane [30]–[32]. As shown in Fig. 2, the expression of these genes was not substantially affected by the stress, suggesting that within this subset of genes, the effect of stress is *var* gene specific.

**Figure 3:**
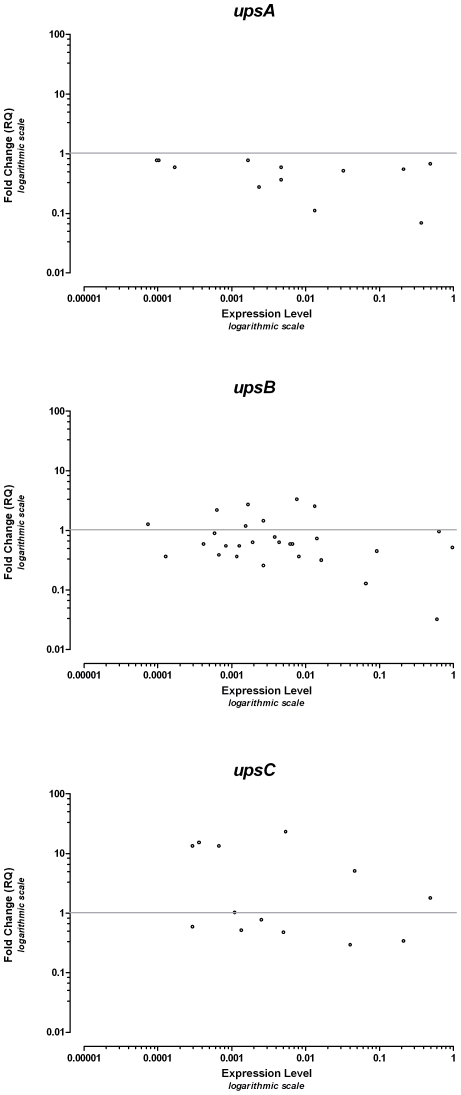
Fold change of *individual var* genes upon exposure to oxidative stress plotted against expression levels. The data was plotted as logarithmic values of RQ versus the base-line logarithmic level of expression of each of the *var* genes. Marks above the horizontal line represent genes whose expression is induced and those below the line represent genes whose expression is reduced. Panels A, B and C show the breakdown to the respective *ups* subtypes.

To study whether the differential effect of stress on *var* gene subtypes is apparent after several generations, the effect of oxidative stress on the expression of 59 individual *var* genes was examined. This was performed under slightly different conditions. Synchronized parasites were exposed to 500 nM tBHP for 6 days and then re-synchronized to ring form. RNA was extracted 10 hours later and qPCR analysis of the expression of each individual *var* gene was performed. In order to analyze whether the changes observed in this set of data correlate with the phenomenon seen when using the general *ups* primers (Fig. 1), we plotted this data as fold-change versus level of expression for each individual *var* gene (Fig. 3). This demonstrates, in agreement with the initial results, that throughout the repertoire of *var* genes the expression of the subtelomeric genes (Fig. 3A, B) are mostly repressed whereas genes of the central region are mostly induced (Fig. 3C).

### ups promoter sequence or chromosomal position?

**Figure 4:**
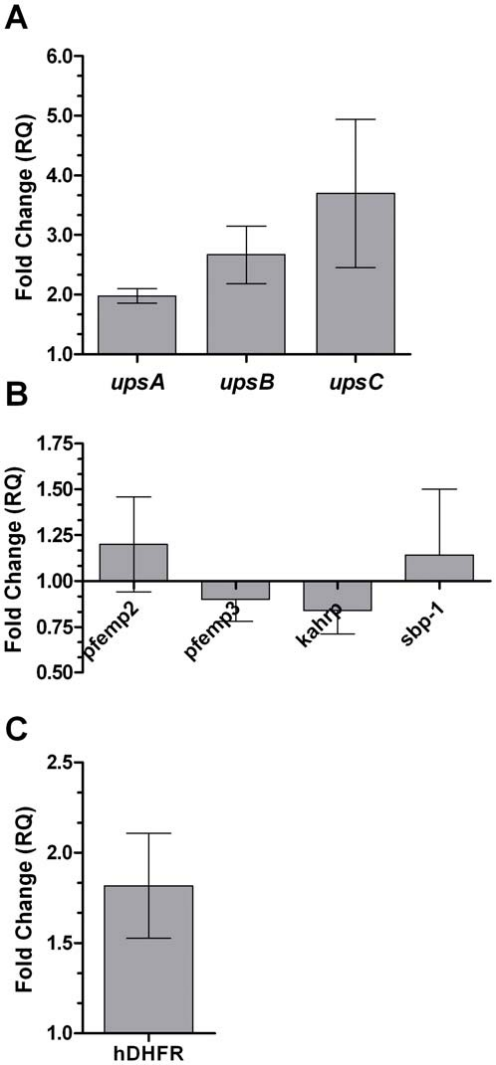
Analysis of expression of *var* and non-*var* exported genes upon exposure to oxidative stress in genetically modified parasites. A. The relative change in expression of the *ups A, B, and C var* subtypes upon exposure to 10 µM *t*BHP in Δ*pfsir2* parasites in relation to unexposed Δ*pfsir2* parasites. B. Analysis of expression of exported non-*var* genes upon exposure to oxidative stress in Δ*pfsir2* parasites. C. Analysis of expression of h*dhfr* – the gene under control of an *upsC* element in the 3D7/upsC transgenic line – upon exposure to 10 µM tBHP. Stress induction, cDNA production and qPCR analysis were performed as described in the legend to Fig. 1. Error bars represent mean of triplicates ± SEM.

We further pondered whether this differential effect on *var* genes is due to the difference in the upstream *sequence* of the subtypes' promoters or whether it is due to their *position* along the chromosome. To address this question, we used the two genetically modified parasite lines described in *Materials and Methods*. We hypothesized that *pfsir2* may act as a leading candidate for involvement in the stress response of *var* genes. In light of this possibility, when repeating the exposure to stress on a *pfsir2* knockout line, a different pattern was observed – stress up-regulated all three subtypes (Fig. 4A). No significant change was observed when examining the other exported genes (Fig. 4B) leading to the conclusion that the *upsA* and *upsB* ***down***-regulation upon stress exposure is a *pfsir2*, hence a positional, dependent process.

To examine whether the stress induced ***up***-regulation of *var* genes is independent of chromosomal positioning we used the 3D7/upsC parasites transfected with the pHBupsC episomal DNA plasmid which caries a gene encoding h*dhfr* driven by an *upsC* promoter. Studying the transcription level of h*dhfr* upon stress exposure was used as a tool to analyze *var* promoter function uncoupled from its chromosomal context. Stress induced the expression of the plasmid's h*dhfr* (Fig. 4C), suggesting that **up**-regulation of *upsC var* genes under stress is also a promoter (sequence) – dependent event.

### Response of var genes upon modulation of pfsir2 activity

The results obtained hitherto implicate *pfsir2* as a crucial factor involved in repression of subtelomeric copies of *var* genes under stress-inducing conditions. It was of interest to further examine the response of *var* gene expression upon isolated modulation of *pfsir2* using well studied chemical agents.

**Figure 5:**
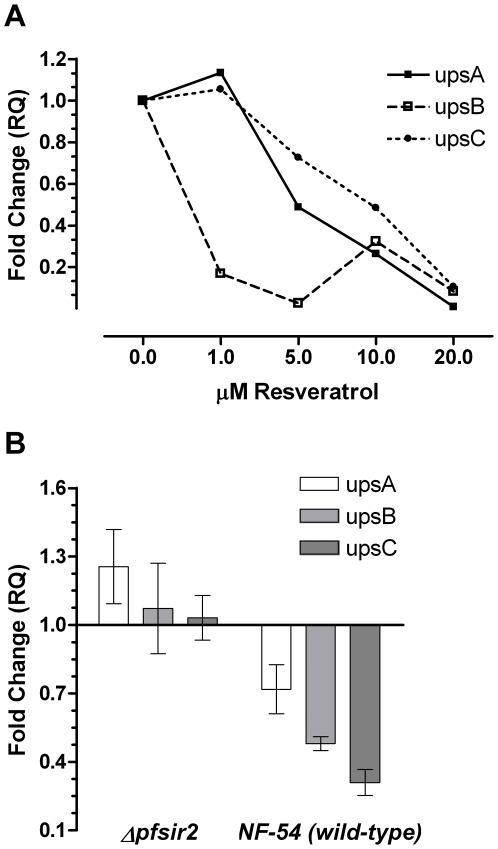
The effect of resveratrol on *var* gene expression. A. Analysis of expression of *var* subtypes upon exposure to RV in wild-type parasites. Synchronized ring stage parasites were exposed for 8 hours to various concentrations of RV. B. Analysis of expression of *var* subtypes upon exposure to 10 µM RV in wild-type and Δpfsir2 parasites. Synchronized ring stage parasites were exposed for 8 hours to 10 µM RV. cDNA production and qPCR analysis were performed as described in the legend to Fig. 1.

To study the effect of *pfsir2* activation we assessed the effect of resveratrol, a polyphenol found in red grapes and peanuts, which was found to activate *sir2* in various eukaryotes [33], [34]. Fig. 5A demonstrates a dose dependent repressive response of all the *var* gene subtypes to an eight hour exposure to resveratrol. This effect is not observed when repeating the exposure on the *pfsir2* knockout line (Fig. 5B), suggesting that the resveratrol induced down-regulation of *var* genes is indeed a *pfsir2* dependent process.

**Figure 6:**
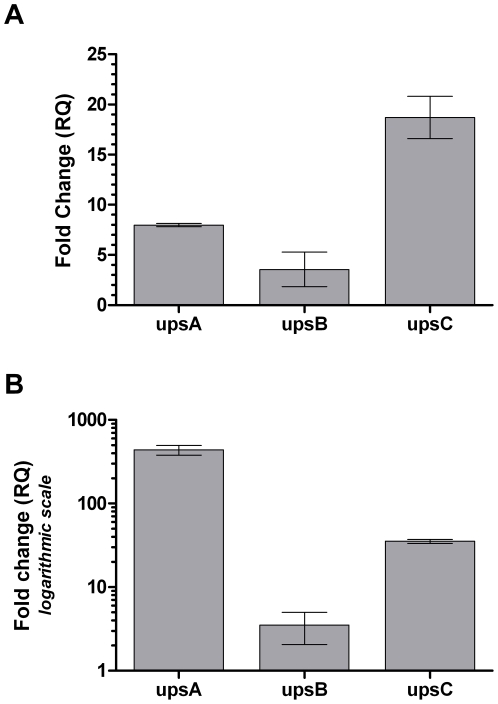
The effect of *pfsir2* inactivation on *var* gene expression. A. Change of expression of *ups* subtypes in NAC exposed wild-type culture in relation to an unexposed culture. Synchronized ring stage parasites were exposed to 5 µM of NAC for 8 hours. B. Relative expression of *ups* subtypes in the *pfsir2* knock-out line in relation to the wild-type line under normal culturing conditions. Note that *y* axis is in logarithmic scale. cDNA production and qPCR analysis were performed as described in the legend to Fig. 1.

We rationalized that if stress-inducing conditions activate *pfsir2* (discussed below), then *protection* from stress might decrease *pfsir2* activity. To study this we used a well-known antioxidant widely used in clinical settings - *N*-acetylcysteine (NAC) - albeit no studies demonstrate that it inactivates sirtuins. When exposing synchronized parasites to an 8 hour pulse of 5 µM NAC, *upsA* and *upsC* sub-types are substantially induced while *upsB* genes are only negligibly up-regulated (Fig. 6A). This pattern is similar to the comparative pattern obtained when examining *var* gene expression in the Δ*pfsir2* line (Fig. 6B), suggesting that NAC indeed inactivates *pfsir2*.

## Discussion

The focus of our study was to examine the effect of stress that the parasite encounters throughout its lifecycle on the expression of *var* genes. The parasite is exposed to oxidative stress in its natural dwellings – the IE. Increased exposure to reactive oxygen species are also found in the pulmonary circulation and possibly when encountering immune cell surveillance. Decreased glucose levels are one of the hallmarks of severe malaria as patients can develop hypoglycemia as low as 50 mg/dl. It is therefore reasonable to assume that the parasite has evolved mechanisms to deal with such forms of metabolic perturbations.

We hypothesized that the differences in upstream promoter sequence and chromosomal location of the *var* genes might influence their expression pattern under stress-inducing conditions. Hence, the qPCR was initially performed with primers which recognize the common upstream promoter regions of the 3 main *ups* subtypes. Upon short exposure to oxidative stress or glucose deprivation, expression of the subtelomeric *upsB* and *upsA* subtypes are repressed while that of the centrally located *upsC* subtype is induced. In order to examine whether the effect is carried over several generations we performed the gene expression analysis after several days of exposure to low-level oxidative stress, this time studying expression of each member of the *var* gene repertoire. Exposure to oxidative stress causes a change in the expression pattern of the *var* genes, with a general trend of inducing the expression of *upsC var* genes while repressing the *upsA* and *upsB* subtypes, corroborating the results obtained with the general *ups* primers. The differential pattern spans all levels of expression – affecting genes of high and low levels of expression. This points to the possibility that the response to stress is achieved in a manner that does not involve the proposed allelic exclusion mechanism(s) and acts as a novel, additional layer of *var* gene regulation. The lack of effect of oxidative stress on a representative group of other exported genes with topological and functional relationships to the PFEMP1 suggests that the stress inducing conditions we examined affect the *var* gene family in a specific and organized manner and not as part of a general transcriptional response of the parasite to stress.

Taken together, these results all point to the fact that there seems to be a certain stability to the *upsC* sub-type as it is preferentially expressed under changes in environmental conditions. Several recently published studies are in accordance with our findings: *a)* Exposure of parasites to febrile temperatures showed an increase in the expression of 5 *var* genes [35], 4 of them belonging to the *upsC* group. *b)* At the initiation of gametogenesis, an event influenced by environmental signals [36], there was a switch from the existing *var* expressed during the asexual stages to a *var* from the *upsC* subtype [37]. *c)* During the period of adaptation of parasites to culture, there was a general down regulation of all *var* genes. However, in those who display *upsA*, *D* and *E* sequences, the rate of down regulation was significantly faster than the genes flanked by *upsB* and *C* [38]. *d)* Studies of switching rates also showed that expression of *var* genes located in the central regions of chromosomes are remarkably stable and that they rarely undergo transcriptional switches in the absence of selection, whereas parasites expressing subtelomerically located *var* genes readily switch them to alternative *var* loci [39].

The phenomenon of the differential effect of stress on centrally-positioned and telomerically-placed *var* genes raises the question whether this is based on sequence of the upstream promoters of the genes or their position along the chromosome. This question was answered by studying the response of *pfsir2* knock-out and 3D7/upsC strains to oxidative stress. These experiments show that the ***up***-regulation of *var* genes upon exposure to stress is most likely a *sequence-dependent* event while ***down***-regulation is probably a *positional-dependent* event, the net effect of both processes results in the differential, positional-dependent pattern observed upon exposure to stress.

We further examined the effect of *pfsir2* on *var* gene expression by modulating its activity thereby adding new insights regarding the regulation of this enzyme on the expression pattern of the *ups* subtypes. Resveratrol, a sirtuin activator, decreased expression of all *var* gene subtypes, regardless of chromosomal position, in a dose- and *pfsir2*-dependent manner. This finding suggests a *pfsir2* modulation over *upsC* genes, a conclusion that is also apparent from the examination of *ups* expression in the Δ*pfsir2* line using qPCR (Fig. 6B) and from previous work studying *var* expression using DNA microarrays in Δ*pfsir2* [23]. Both experiment show *upsC* gene up-regulation in the absence of the TPE. However, in a chromatin immuno-precipitation (ChIP) study preformed by Scherf and colleagues [24] using anti-*pfsir2* antibodies, *pfsir2* binding was demonstrated to occur in telomeric regions with repression of *upsB* expression while it was also clearly shown to *not* bind or have a direct effect on *upsC var* genes (*upsA* genes were not studied). This discrepancy can be resolved by predicting the existence of additional layer(s) of control, a likely possibility being a transcription factor under direct control of *pfsir2* which exclusively activates *upsC var* genes. The activity of such a transcription factor on *upsC* will not be apparent when utilizing a ChIP assay while it's un-repressed effect will easily be demonstrated when examining expression levels of *upsC var* genes in a *pfsir2* knock-out.

In contrast to the ***activation*** of *pfsir2* using resveratrol, experimentation with NAC presents a *var* expression pattern consistent with *pfsir2* ***deactivation*** as it is similar to the pattern of basal *var* expression observed in the Δ*pfsir2* line – prominent up-regulation of *upsA* and *upsC var* genes with minimal effect on *upsB* (Fig. 6B). Review of the literature shows that a relationship between NAC and sirtuin inhibition was never studied, however such a relationship seems plausible as NAC, an antioxidant, could (directly or indirectly) reduce NAD^+^ to NADH thus resulting in NAD^+^ depletion. As *pfsir2* is a NAD^+^ dependent enzyme [40], this would result in decreased activity of the enzyme.

**Figure 7:**
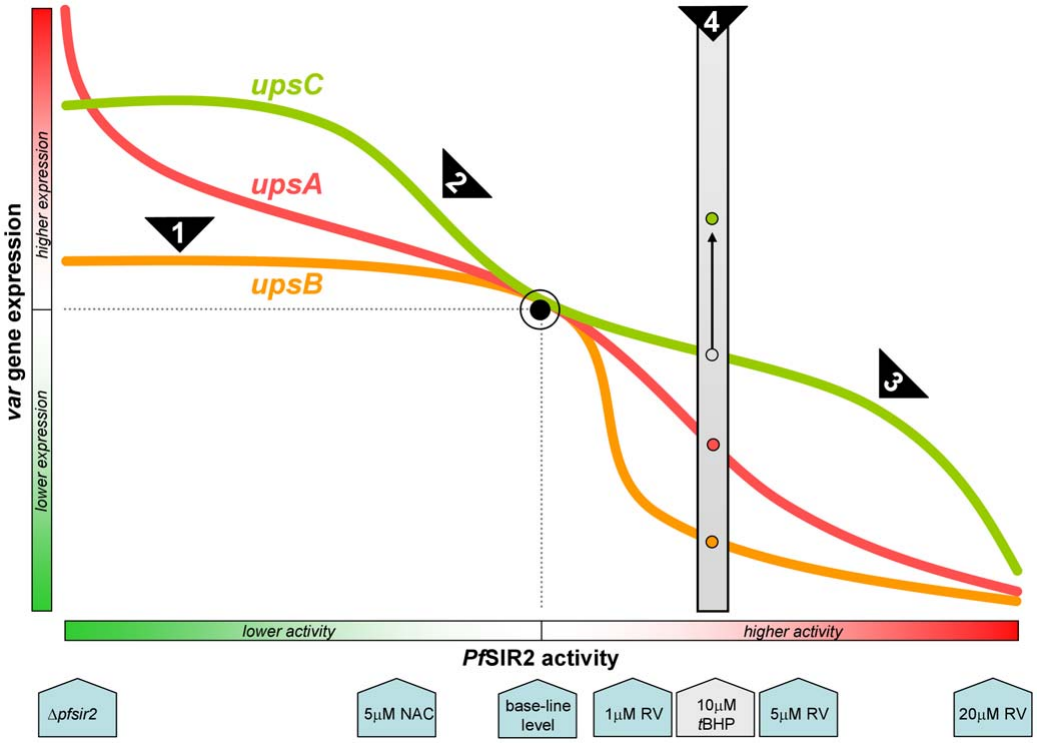
Schematic representation of the proposed theory of *var* modulation by *pfsir2*. This schema summarizes the main findings and theories put forward in this work. *pfsir2* plays a dominant role in *var* gene regulation, its level of activity differentially determining the extent of *var* gene expression. *x* axis represents *pfsir2* activity, *y* axis represents relative *var* expression. This figure does not represent quantitative relationships. See text for further explanation. Arrowheads at bottom row show the main experiments involved in the establishment of this theory.

When incorporating all the data obtained in this study, an integrated picture of the *pfsir2*-dependent orchestrated response of *var* genes to changes in the external environment can be built (schematically presented in Fig. 7):

When *pfsir2* is ***inactivated*** it releases the sub-telomeric *var* genes from its inhibitory effect. However, *upsB var* genes are not up-regulated *de facto* due to the existence of the additional *upsB*-specific HDAC (black arrow 1 in Fig. 7). As for *upsC*, this subtype is not under direct control of *pfsir2*, yet its expression is affected by a predicted transcription factor which is under *pfsir2* control. When *pfsir2* activity is reduced, the proposed transcription factor is freed of repression thus allowing increased expression levels of *upsC* genes (black arrow 2 in Fig. 7).

When *pfsir2* is ***activated***, it binds to the telomere-associated *rep20* sequences and propagates inhibition into the chromosome. The end effect of activated *pfsir2* is dependent on the intensity of activation. At low levels of *pfsir2* activation, the inhibition will be most prominent on the *upsB* subtype *var* genes as they are closest to the telomeres. At higher levels of *pfsir2* activation, the inhibitory effect of *pfsir2* increases as it propagates deeper into the chromosomal sequence to encompass the *upsA var* genes. When *pfsir2* activity is highest, it represses the putative *upsC* transcription factor thus resulting in the addition of *upsC* genes to the inhibitory effect of *pfsir2* (black arrow 3 in Fig. 7). In other words, *pfsir2* activation has a “dose-responsive” effect – increasing levels of *pfsir2* activity first affect *upsB* genes, followed by *upsA* genes and lastly *upsC* genes. Careful examination of the data obtained with resveratrol (Fig. 5A) show precisely such a dose-dependent pattern: At 1 µM only *upsB* is down-regulated (>80%) with no effect on the other sub-types, at 5 µM *upsA* is also down regulated (>50%), and at 20 µM all three subtypes are equally repressed.

This model also contributes to the explanation of the response of *var* genes to stress exposure (black arrow 4 in Fig. 7): Stress inducing conditions would cause induction of *var* genes regardless of their subtype classification or their chromosomal position. However, stress also activates *pfsir2* (in *S. cerevisiae* the *sir2* gene is activated upon exposure to calorie restriction and oxidative stress [41]), increasing histone deacetylation in the telomeric region, thereby repressing genes positioned in the vicinity. Upon exposure to stress the sum effect of these two processes results in the observed effect of increased expression of centrally positioned *var* genes free of direct *pfsir2* influence, and repression of *var* genes that are under control of *pfsir2 i.e.* the subtelomerically positioned genes.

Our results with resveratrol and NAC have obvious scientific and clinical applications. From a clinical standpoint, further research is warranted to determine whether, *in-vivo*, resveratrol-induced down-regulation of *var* genes results in decreased binding of IEs to the endothelium. If so, this substance can potentially be used as a safe, inexpensive yet powerful adjuvant antimalarial therapy that can alleviate the clinical complication of malaria caused by the IE sequestration in the microvasculature – namely cerebral and placental malaria. As for NAC, the dominant up-regulation of *upsC* subtypes this compound causes may possibly confer protection to the host as expression of this subtype is correlated to milder outcome of disease and lower incidence of cerebral malaria. Indeed, a clinical trial conducted on human patients suffering from severe malaria using NAC as an adjuvant therapy demonstrated improved outcomes of these patients [42].

To summarize, this study sheds new light on the regulatory processes of *var* gene expression. It is apparent that stress-inducing conditions affect *var* genes in a differential, positional dependent manner. Epigenetic machinery is involved in the establishment of this pattern, and manipulation of its mechanism results in distinct patterns of *var* gene expression. This can be harnessed for development of novel *var*-based treatments to alleviate the severe consequences of this disease.
